# Inhibition of Nitric Oxide Production in BV2 Microglial Cells by Triterpenes from *Tetrapanax papyriferus*

**DOI:** 10.3390/molecules21040459

**Published:** 2016-04-07

**Authors:** Namki Cho, Eun Hye Moon, Hyun Woo Kim, Jaewoo Hong, John A. Beutler, Sang Hyun Sung

**Affiliations:** 1 College of Pharmacy and Research Institute of Pharmaceutical Science, Seoul National University, Seoul 151-742, Korea; cnamki@naver.com (N.C.); arsinoe@snu.ac.kr (E.H.M.); kimkami2@snu.ac.kr (H.W.K.); 2 Molecular Targets Laboratory, Center for Cancer Research, National Cancer Institute, Frederick, MD 21702, USA; beutlerj@mail.nih.gov; 3 Mouse Cancer Genetics Program, Center for Cancer Research, National Cancer Institute, Frederick, MD 21702, USA; jaewoo.hong@nih.gov

**Keywords:** *Tetrapanax papyriferus*, triterpenes, BV2 microglial cells, NO, COX-2

## Abstract

It is well known that activated microglia produce nitric oxide (NO), which has an important role in the pathophysiology of several neurodegenerative diseases such as Alzheimer’s disease. In the course of searching for novel therapeutic agents from medicinal plants against neuroinflammatory diseases, the methanolic extract of *Tetrapanax papyriferus* was found to have significant NO inhibitory activity in lipopolysaccharide (LPS)-stimulated BV2 microglia cells. Nine oleanane-type triterpenes, including two new compounds, epipapyriogenin C-3-*O*-β-d-glucopyranoside (**6**) and 11-*O*-butylpapyrioside LIIc (**9**), were isolated from the leaves and stems of *Tetrapanax papyriferus*. The structures of these compounds were elucidated with 1D- and 2D-NMR and MS data. Among these Δ^11,13^ oleanane-type triterpenes, compound **3** showed significant NO inhibitory activity in BV-2 cells, reducing the LPS-induced expression of COX-2 and pro-inflammatory cytokines such as TNF-α and IL-6. Compounds **7** and **9** also showed NO inhibitory activities among the Δ^12^ oleanane-type triterpene saponins. These results show that oleanane-type triterpenes isolated from *T. papyriferus* could be a potential natural resource of NO inhibitors used in the treatment of neurodegenerative disorders.

## 1. Introduction

Chronic neuroinflammation induces neurodegenerative diseases such as Alzheimer’s disease (AD) and Parkinson’s disease (PD) [1]. Neuroinflammation is associated with activated microglia, which are CNS-stationed immune cells. Activated microglia induced by inflammation-inducing materials such as LPS release an abundant amounts of reactive oxygen species (ROS), inducible nitric oxide synthase (iNOS) and cyclooxgenase-2 (COX-2) as well as tumor necrosis factor-α (TNF-α), interleukin (IL)-1β, IL-6 and nitric oxide (NO) [2]. Nitric oxide (NO) is well known as a small membrane-permeable gas which is produced via the oxidation of L-arginine by nitric oxide synthases [3]. Although normal levels of NO can act as a neuromodulator at synaptic junctions, high levels of NO secreted by activated microglia induce oxidative stress and neuron destruction contributing to neurodegenerative disorders [2,3]. In other words, while inflammation is crucial, it has been associated with normal aging as well as age-related neurodegenerative diseases [4]. Therefore, we screened for compounds from medicinal plants that could modulate NO production.

During our search for new regulators of neuroinflammation from natural products, the methanolic extract of the leaves and stems of *Tetrapanax papyriferus* (Hook) K. Koch (Araliaceae) showed significant inhibitory activity against NO production (9.3% ± 6.5% of the control at a concentration of 100.0 μg/mL)*. T. papyriferus* is an evergreen shrub native to Taiwan and China and traditionally used as a herbal medicine [5]. Several studies have reported that the extract of *T. papyriferus* has anti-inflammatory, anti-thrombin and anti-hepatotoxic activities [5]. Regarding pharmacological studies on triterpene saponins isolated from this plant, it has been reported that several papyrogenins showed remarkable antihepatotoxic effects in primary hepatocytes and anti-HIV activity in the T cell line H9 [6]. However, to the best of our knowledge, there have been no reports on anti-neuroinflammatory activity of *T. papyriferus* against LPS-induced NO production in BV2 microglial cells. Some of the varied saponins in *T. papyriferus* stems and leaves are more difficult to isolate and have complex chemical structures that are difficult to identify, hindering the study of their structure and function [7]. In the present study, we identified the bioactive constituents of *T. papyriferus* stems and leaves which have NO inhibitory activities in LPS-activated BV2 microglial cells. In addition, this paper describes the structural elucidations of two new oleanane-type triterpenoids and suggests the importance of oleanane-type triterpenoids in the inhibition of nitric oxide in BV2 cell lines. 

## 2. Results and Discussion

### 2.1. Isolation and Characterization of Oleanane-Type Triterpenoids

**Figure 1:**
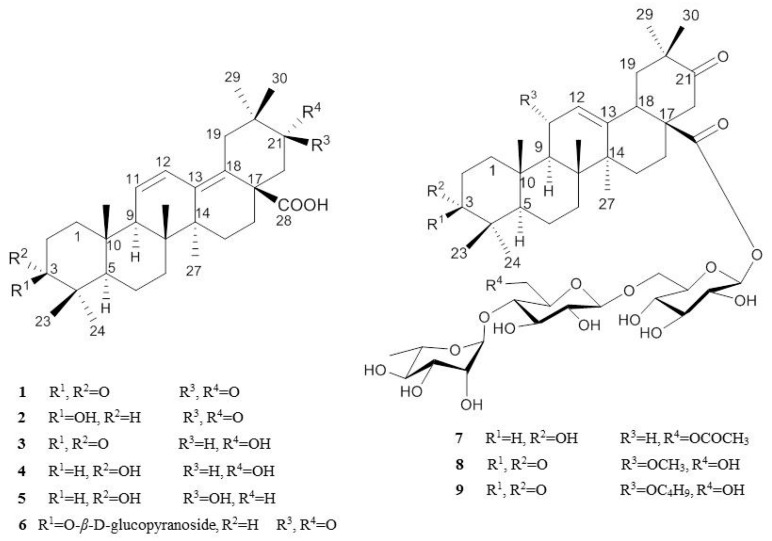
Isolated compounds from *T. papyriferus*.

The ^1^H- and ^13^C-NMR spectral data of compound **1** showed the characteristic signals of Δ^11,13^ type triterpenes, including two oxo groups, a carboxylic carbon and four olefinic carbons (Figure 1). The HMBC correlations showed that the two oxo groups were present at C-3 and C-21, respectively, and a carboxylic group was present at C-28. Based on the above information, compound **1** was determined to be papyriogenin A [7].

**Figure 2:**
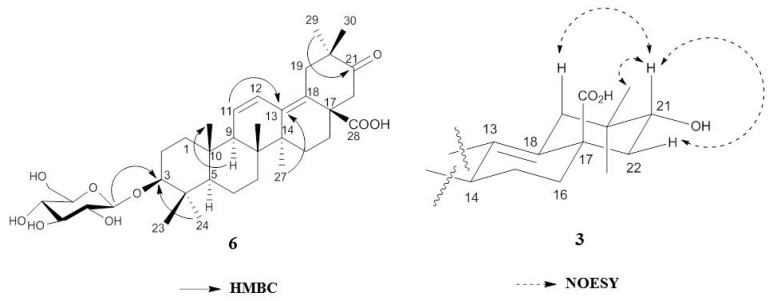
HMBC correlation of compound **6** and NOESY correlation of compound **3**.

The spectra of compound **2** were similar to those of compound **1**, except for the signal at δ_H_ 3.50 (1H, dd *J* = 10.40 Hz, 6.20 Hz, H-3) and δ_C_ 78.0 (C-3) (Table S1). This carbon signal indicated that the oxo group at C-3 was replaced with a β-hydroxyl moiety because compounds with an α-hydroxyl moiety exhibit a lower field signal (*ca.* 75.0 ppm) [7]. Based on the above information, compound **2** was determined to be epipapyriogenin C [7]. The ^1^H- and ^13^C-NMR spectra of compound **3** showed similar patterns of those of compound **1**, except for the signal at δ_H_ 4.11 (1H, dd, H-21β) having a cross peak with δ_C_ 73.9 (C-21) in the HSQC analysis. The NOESY spectra revealed correlations between 30-CH_3_ and H-21, H-21 and H-22; thus, the absolute structure of compound **3** was identified (Figure 2) and with the above observed spectroscopic data, confirmed as papyriogenin D [8]. The spectra of compound **4** were similar to those of compound **3**, except for the signal at δ_C_ 75.3 (C-3) (Table S3). In the carbon spectrum, this signal indicated that the oxo group at C-3 was replaced with a hydroxy group. Based on the above information as well as by comparison with literature data [8,9], compound **4** was determined to be papyriogenin E. The ^1^H- and ^13^C-NMR spectra of compound **5** showed similar patterns to those of compound **4**, except for the signal at δ_H_ 3.51 (1H, dd, H-21α) having a cross peak with δ_C_ 74.4 (C-21) in the HSQC analysis. With the above observed spectroscopic data, compound **5** was confirmed as papyriogenin E′ [8,9].

Compound **6** was newly isolated and obtained as a white amorphous powder (${\left[\alpha \right]}_{D}^{25}$ − 7.6; *c* = 0.1, MeOH). The molecular formula C_36_H_54_O_9_ was established by HRMS (*m/z* 631.3845 [M − H]^−^) and the ^13^C-NMR spectrum. The ^1^H- and ^13^C-NMR spectra of compound **6** were similar to those of compound **2**, except for the signals of the anomeric carbon (δ_C_ 107.0) (C-1′) and glucose groups. In the HMBC experiment, the correlation between the anomeric proton (δ_H_ 4.95) and C-3 (δ_C_ 88.8) indicated that the hydroxy group at C-3 was replaced with a glucosyl group. Based on the above information as well as by comparison with the literature [10], compound **6** was determined to be epipapyriogenin C-3-*O*-β-d-glucopyranoside.

Compound **9** was newly isolated as a dark brown syrup (${\left[\alpha \right]}_{D}^{25}$ 114.6; *c* = 0.1, MeOH). Its molecular formula was established as C_52_H_82_O_19_ by HRMS (*m/z* 1009.5394 [M − H]^−^) and the ^13^C-NMR spectrum. The ^1^H- and ^13^C-NMR spectral data of compound **9** exhibited three anomeric protons, including two glucosyl and one rhamnosyl group and the characteristic signals of Δ^12^ type triterpenes, including two oxo groups, a carboxylic carbon and two olefinic carbons. The HMBC correlations indicated that the two oxo groups were present at C-21 and C-3, respectively, and a carboxylic group was present at C-28 and signals of a butoxy moiety at C-11. With the above observed spectroscopic data, compound **9** was confirmed as 11-*O*-butylpapyrioside LIIc. The ^1^H- and ^13^C-NMR spectra of compound **8**, papyrioside LII, showed similar patterns to those of compound **9**, except for the signals of a methoxy moiety at C-11 (Table 1). The ^13^C-NMR spectra of compound **7** were similar to those of compound **8** except for the signals at δ_C_ 26.3 (C-2), 75.1 (C-3), δ_C_ 47.8 (C-9), δ_C_ 23.8 (C-11) and δ_C_ 63.6 (C-6′′) (Table S4). In the HMBC experiment, this signal indicated that the oxo group at C-3 was replaced with hydroxyl group; α-methoxy moiety at C-11 was substituted with a hydrogen, and a hydroxyl group at C-6′′ was replaced with the acetoxy group. Based on the above information, compound **7** was determined to be papyrioside LF [8,11,12].

### 2.2. NO Inhibitory Effects of Oleanane-Type Triterpenoids

Inhibition of NO production in LPS-induced BV-2 cells is considered an effective treatment for inflammation in the CNS. It is well known that BV-2 cells, an immortalized murine microglial cell line, are activated when stimulated and subsequently, release iNOS and finally NO which is similar to the process of microglial action *in vivo*. Thus, BV-2 cells were used as a screening tool when searching for therapeutic agents against neuroinflammation in natural products.

The NO inhibitory activities of the isolated compounds were evaluated in LPS-stimulated BV-2 cells (Table 2). The cytotoxicity of these compounds was measured by the MTT assay. Compounds **1**–**6**, Δ^11,13^ oleanane-type triterpenes, showed cytotoxicity at 50.0 µM, whereas the saponins **7**–**9** showed little toxicity at 100.0 μM. Thus, the NO inhibitory activity of compounds **1**–**6**, Δ^11,13^ oleanane-type triterpenes, was evaluated in LPS-stimulated BV-2 cells at a concentration ranging from 1.0 to 10.0 µM. Among these, compounds **1**, **3**, **4** and **5** but not compound **2** showed significant NO inhibitory activities in BV-2 cells. Of note, the NO inhibitory activity of compound **5** (6.9% at 10.0 µM, IC_50_ = 3.4) was superior to that of compound **2** (41.4% at 10 µM, IC_50_ = 4.8) despite the structural similarity. Compound **3** (9.2% at 5.0 µM, IC_50_ = 2.9) also had a higher NO inhibitory activity than that of compound **1** (67.2% at 5.0 µM, IC_50_ = 6.3). 

Based on our results, the degree of the substitution at C-3 or C-21 likely affected the NO inhibitory activity. In addition, in our *in vitro* system, compound **5** (10.5% at 5.0 µM, IC_50_ = 3.4), which has a β-hydroxyl moiety at C-21, had a more potent NO inhibitory activity than that of compound **4** (56.3% at 5.0 µM, IC_50_ = 5.9) which has an α-hydroxyl moiety. The inhibitory activities of compounds **7**–**9** against NO production were evaluated in LPS-stimulated BV-2 cells at a concentration ranging from 10.0 to 100.0 μM (Table 2). Among these Δ^12^ oleanane-type triterpene saponins, compounds **7** and **9** showed significant NO inhibitory activities in BV-2 cells. Specially, compound **9** (14.3% at 100.0 µM, IC_50_ = 42.1) with a butoxy group at C-11 had a slightly higher NO inhibitory activity than that of compound **8** (53.9% at 100.0 µM, IC_50_ > 100.0) with a methoxy group at C-11.

It has been assumed that the potent inhibitory activities of oleanane-type triterpenes are associated with the degree of unsaturated double bonds and substituted moieties [13,14]. In agreement with these studies, here, we clarified this assumption by investigating the NO inhibitory activities of oleanane-type triterpenes isolated from *T. papyriferus*. Our study showed that the replacement of substituents at C-3 and C-21 are important determinants of NO inhibitory activity in LPS-induced BV2 cells.

**Figure 3:**
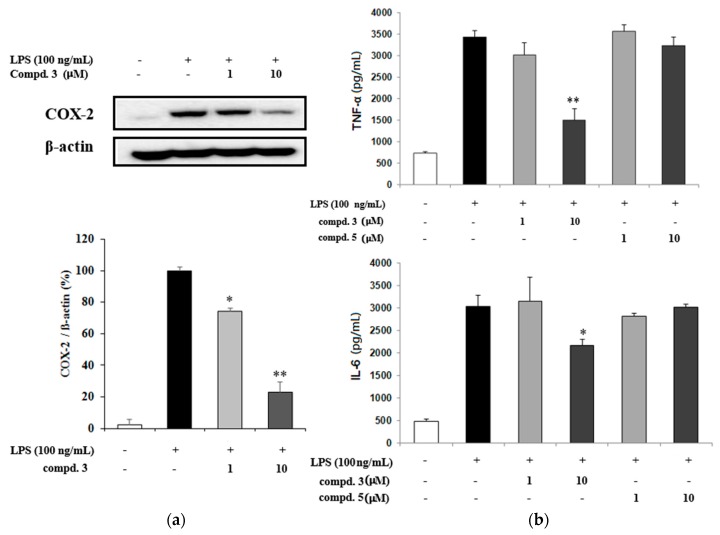
(**a**) The expression of COX-2 in LPS-activated BV2 microglia. The relative expression levels of COX-2 were determined by densitometry and normalized by actin; (**b**) The effects of compounds **3** and **5** on TNF-α and IL-6 secretion in LPS-activated BV2 microglia. Results represent the mean ± SD of three independent experiments. Results differ significantly from the LPS-treated, * *p* < 0.05, ** *p* < 0.01. +: sample treated, −: no treated.

Western blot analysis was done for the expression of COX-2 protein and proinflammatory mediators to evaluate the effects of compound **3** which showed a potent NO inhibitory activity in the BV2 cells. The results show that compound **3** inhibited COX-2 protein expression which suppressed TNF-α and IL-6 production (Figure 3).

To the best of our knowledge, this is the first report on the NO inhibitory activities of oleanane-type triterpenes in BV2 microglia cells. Their different degrees of NO inhibitory activities *in vitro* could be structure-dependent. There have been many considerable attention given to *T. papyriferus* in the course of searching for the neuroprotective agents, since triterpenoids are believed to be closely linked with some important neurodegenerative diseases such as, Alzheimer’s disease and Parkinson’s disease [15,16,17]. Our findings provide a preliminary indication that the oleanane triterpenes have potential anti-neuroinflammatory effects, and further research into *T. papyriferus* and its constituents would be very valuable. Taken together, although further studies need to unravel the specific cellular and molecular mechanisms, it could be postulated that the oleanane-type triterpenes of *T. papyriferus* have potential as a natural resource for NO inhibitors that could be used in the treatment of some neurodegenerative disorders.

## 3. Materials and Methods

### 3.1. Plant Materials

Leaves and stems of *T. papyriferus* (3.0 kg) were collected at the Medicinal Plant Garden, College of Pharmacy, Seoul National University, Goyang, Korea, in May 2011 and then air-dried. They were authenticated by Dr. Tae Jin Yang, a professor at Seoul National University. A voucher specimen (SNU-1122) has been deposited in the Herbarium of the Medicinal Plant Garden of the College of Pharmacy, Seoul National University.

### 3.2. Extraction and Isolation

In the present study, the dried leaves and stems of *T. papyriferus* were extracted with 80% MeOH in an ultrasonic apparatus. After removal of the solvent *in vacuo*, the 80% MeOH extract was successively partitioned into *n*-hexane, EtOAc, and *n*-BuOH fractions, respectively. Among these fractions, the EtOAc and *n*-BuOH fractions showed NO inhibitory activity in LPS-activated BV-2 cells (17.0% at 100.0 μg/mL and 39.0% at 100.0 μg/mL, respectively), and were subjected to repeated column chromatography and HPLC to yield newly isolated compounds **6** and **9**, along with seven known compounds.

The EtOAc fraction was subjected to silica gel column chromatography (CC) (55.0 cm × 7.5 cm) eluted with mixtures of CHCl_3_–MeOH = 30:1, 10:1, 5:1, 1:1, MeOH; *v*/*v* to yield ten fractions (E1–E10). E2 and E8 were further separated into ten (E2-1~E2-10) and four (E8-1~E8-4) fractions respectively on ODS gel CC (30.0 cm × 5.0 cm) eluted with mixtures of MeOH–DDW = 7:3, 8:2, 9:1. Compound **5** (3.0 mg), **3** (20.0 mg), and **1** (12.0 mg) were isolated from E2-7, E2-9, and E2-10 respectively by recrystallization (MeOH). Compounds **2** (23.1 mg) and **4** (5.2 mg) were purified from E2-8 by ODS silica gel HPLC using ACN–H_2_O (6:4, 10 mL/min). E8-2 was further separated into three fractions (E8-2-1~E8-2-3) using Sephadex LH-20 with MeOH and E8-4 was subjected to silica gel CC (20 cm × 5 cm) eluted with mixtures of CHCl_3_–MeOH = 7:1, 3:1, 1:1, MeOH to yield compound **7** (228.1 mg) and E8-4-11 was further purified by recrystallization (MeOH) to yield compound **6** (3.1 mg). The BuOH fraction was separated into four fractions (B1–B4) using HP-20 resin eluted with a gradient of aqueous MeOH (MeOH–H_2_O = 1:4, 5:5, 4:1). B4 was subjected to ODS gel CC (30.0 cm × 5.0 cm) eluted with mixtures of MeOH–DDW = 6:4, 8:2, 9:1, and MeOH to yield compound **8** (620.1 mg) and compound **9** (300.0 mg).

### 3.3. Cell Line

BV-2 cell line was provided by Dr. Sun-Yeou Kim at Kyunghee University (Suwon, Korea) and maintained in DMEM supplemented with 10% heat-inactivated fetal bovine serum, 100 IU/mL penicillin and 100 µg/mL streptomycin at 37 °C in a humidified incubator containing 5% CO_2_ gas.

### 3.4. Cell Line Culture

For the assay, BV2 microglia cells (3 × 10^5^ cells/well in 96 well plates) were treated with test samples for 1 h before exposure to 100 ng/mL of LPS. BV-2 cells were incubated with 2 mg/mL of MTT for 1 h. Reduction of MTT to formazan was assessed in an ELISA plate reader at 540 nm. Data were expressed at percent cell viability relative to control cultures as the mean of three independent experiments. For western blot analysis, a mixture of sample loading buffer (Biosaesang Co., Seoul, Korea) and 30 μg of tissue protein was boiled at 100 °C for 10 min. Denatured proteins were separated by 14% polyacrylamide gel electrophoresis for 2–3 h at 100 V and transferred to a 0.2 μm nitrocellulose membranes for 1.5 h at 100 V. Membranes were then washed 3 times for 10 min in 0.1% Tween-20 PBS between each of the following steps: 1 h block in 5% milk and over-night incubation at 4 °C with primary antibodies (COX-2 and β-actin). The immunoreactive bands were visualized by using secondary antibodies and an ECL chemiluminescence detection kit (Amersham Biosciences, Frederick, MD, USA).

### 3.5. NO Assay and TNF-α and IL-6 ELISA

After 24 h incubation with test samples and LPS, nitrite in culture media was measured to assess NO production in BV2 cells using Griess reagent. In 96 well plate, 100 µL of sample aliquots were mixed with 100 µL of Griess reagent (1% sulfanilamide, 0.1% naphtylethylenediamine dihydrochloride in 2% phosphoric acid) and incubated at room temperature for 15 min. Culture medium was collected, and the concentration of secreted TNF-α and IL-6 was determined using ELISA kits from R & D Systems (Minneapolis, MN, USA) following the manufacturer’s instructions.

### 3.6. Statistical Analysis

All data were expressed as means ± S.D. The evaluation of statistical significance was determined by an “one-way ANOVA” test using computerized statistical package, with *p* < 0.05 *, *p* < 0.01 ** and *p* < 0.001 *** considered to be statistically significant.

## Figures and Tables

**Table 1:** ^1^H-NMR spectral data of compounds **6** and **9**.

Position	6 ^a^		9 ^a^	
	δ_H_ (*J* in Hz)	δ_C_	δ_H_ (*J* in Hz) δ_C_	
1a	1.86 m	38.1	2.62 m	34.5
1b			2.42 m	
2a	2.33 m	26.3	2.19 m	40.4
2b	1.90 m		1.76 m	
3	3.43 dd (11.94, 4.56)	88.8		216.3
4		39.5		47.7
5	0.83 d (11.94)	55.2	1.34 m	55.5
6	1.56 m, 1.38 m	18.3	1.33 m	19.6
7	1.24 m	32.6	1.33 m	32.8
8		41.0		38.0
9	1.99 s	54.6	1.86 m	52.6
10		36.6		43.0
11	5.75 d (10.74)	127.5	4.07 m	76.3
12	6.58 d (10.56)	126.0	5.83 m	124.2
13		137.6		145.6
14		42.2	42.0	
15a	2.34 m	26.5	2.12 m	28.0
15b	2.23 m		1.04 m	
16a	2.24 m	32.1	2.10 m	25.6
16b	1.70 m		1.79 m	
17		50.2		45.4
18		132.2	3.67 m	40.8
19a	2.80 m	40.5	3.13 m	46.2
19b	2.60 d(14.70)		2.55 m	
20		46.0	50.8	
21		213.8	212.0	
22a	3.30 d (14.22)	50.4	2.21 m	47.2
22b	2.48 d (14.22)		1.73 m	
23	1.33 s	27.9	1.13 s 26.4 1.04 s 21.4	
24	1.00 s	16.5		
25	0.93 s	18.3	1.10 s 16.5	
26	0.95 s	16.9	1.09 s 18.9	
27	1.12 s	20.2	1.04 s	25.0
28		185.0		173.9
29	1.15 s	24.8	1.17 s	24.5
30	1.12 s	25.7	1.22 s	25.2
1′	4.95 s	107.0	4.95 6.21 m	96.0
2′	4.06 m	75.7	4.28 m	73.8
3′	4.26 m	78.7	4.18 m 78.5	
4′	4.24 m	71.8	4.26 m	70.7
5′	4.04 m	78.3	3.86 m	75.1
6′a	4.61 m	63.0	4.66 m	69.3
6′b	4.43 dd (11.46, 5.04)		4.26 m	
1′′			4.91 m	104.9
2′′			3.90 m	74.6
3′′			3.61 m	77.0
4′′			4.33 m	78.2
5′′			4.08 m	77.9
6′′a			4.14 m	61.2
6′′b			4.02 m	
1′′			5.78 m	102.6
2′′′			4.49 m	72.6
3′′′			4.62 m	72.4
4′′′			4.08 m	73.6
5′′′			4.88 m	70.2
6′′′			1.65 d (5.94)	18.4
OBu			3.56 m, 3.24 m	66.7
			1.47 m, 1,33 m	32.8
			1.32 m	19.8
			0.81 m	13.9

^1^H- and ^13^C-NMR data were measured at ^a^ 600 MHz in pyridine-*d*_5_.

**Table 2:** NO inhibitory effects of compounds **1**–**9** isolated from *T. papyriferus* on LPS-activated BV2 microglia cells.

	**1.0 μM**		**5.0 μM**		**10.0 μM**	
	**Nitrite (%)**	**Viability (%)**	**Nitrite (%)**	**Viability (%)**	**Nitrite (%)**	**Viability (%)**
**1**	83.7 ± 9.1	83.5 ± 6.1	67.2 ± 11.4	81.4 ± 4.9	6.7 ± 2.3 ***	75.2 ± 7.2
**2**	85.9 ± 13.5	79.8 ± 6.7	48.4 ± 9.3	78.1 ± 2.5	41.4 ± 8.5 *	76.1 ± 5.5
**3**	85.3 ± 8.4	83.2 ±7.3	9.2 ± 5.3 ***	66.8 ± 8.1	5.4 ± 2.4 ***	64.0 ±7.1
**4**	79.6 ± 5.5	102.0 ± 0.6	56.3 ± 6.8	106.0 ± 4.6	11.9 ± 4.2 **	87.6 ± 1.8
**5**	92.0 ± 4.4	97.7 ± 5.1	10.5 ± 3.6 ***	79.7 ± 10.6	6.9 ± 5.0 ***	77.9 ± 4.2
**6**	97.2 ± 3.9	88.1 ± 1.5	83.3 ± 9.3	92.0 ± 5.1	73.4 ± 9.2	88.5 ± 6.3
	**10.0 μM**		**50.0 μM**		**100.0 μM**	
	**Nitrite (%)**	**Viability (%)**	**Nitrite (%)**	**Viability (%)**	**Nitrite (%)**	**Viability (%)**
**7**	69.2 ± 8.9	97.9 ± 8.2	38.4 ± 1.1 *	92.7 ± 6.8	15.1 ± 6.9 **	93.3 ± 5.1
**8**	95.4 ± 3.8	93.1 ± 7.5	87.4 ± 7.0	96.8 ± 5.6	53.9 ± 7.0	90.0 ± 4.5
**9**	103.5 ± 3.8	88.2 ± 1.7	38.4 ± 9.9 *	88.5 ± 7.1	14.3 ± 6.7 **	92.0 ± 5.5
	29.7 ± 2.4 *	89.9 ± 6.6	17.5 ± 3.4 **	90.4 ± 7.4	14.4 ± 3.4 **	95.5 ± 4.3

NO production (NP) of control and LPS-treated cultures were 1.2 ± 0.3 µM and 19.8 ± 1.9 µM, respectively. Relative NO production (%) was calculated as (NP of sample treated-NP of control)/(NP of LPS-treated-NP of control) × 100 (%). Results represent the mean ± SD of three independent experiments. LPS-stimulated value differs significantly from control at a level of *p* < 0.001. Results differ significantly from the LPS-treated, * *p* < 0.05, ** *p* < 0.01, *** *p* < 0.001.

## Supplementary Materials

Supplementary materials can be accessed at: http://www.mdpi.com/1420-3049/21/4/459/s1.
