# Supplementary material for: Inhibition of Nitric Oxide Production in BV2 Microglial Cells by Triterpenes from Tetrapanax papyriferus
Source: Molecules. 2016 Apr 7;21(4):459. doi: 10.3390/molecules21040459 (PMC6273476; doi:10.3390/molecules21040459)
Supplement: Supplementary file 1 [file molecules-21-00459-s001.pdf]

Supplementary Materials: Inhibition of Nitric Oxide Production in BV2 Microglial Cells by Triterpenes from *Tetrapanax papyriferus*

Namki Cho, Eun Hye Moon, Hyun Woo Kim, Jaewoo Hong, John A. Beutler and Sang Hyun Sung

Table S1. ^{1}H-NMR spectral data of compounds **1**–**5**.

Position	**1** [a]	**2** [b]	**3** [c]	**4** [c]	**5** [a,d]
			**δH (*J* in Hz)**		
1a	2.01 m	1.92 m	1.99 m	1.87 m	1.61 m
1b	1.42 m	1.12 m	1.42 m	1.72 m	
2a	2.60 m	1.93 m	2.59 m	2.08 m	2.00 m
2b	2.49 m		2.48 m	1.83 m	1.61 m
3		3.50 dd (10.40, 6.20)		3.64 br s	3.42 t (2.76)
4					
5	1.42 m	0.95 dd (11.52, 1.56)	1.42 m	1.82 m	1.35 m
6	1.45 m	1.66 m	1.42 m	1.54 m, 1.43 m	1.48 m
7	1.32 m	1.38 m	1.29 m	1.35 m	1.32 m
8					
9	2.09 m	2.10 m	2.08 m	2.28 m	2.07 m
10					
11	5.78 d (10.08)	5.89 dd (10.52, 1.28)	5.71 d (10.25)	5.87 d (10.45)	5.70 dd (10.56, 1.86)
12	6.71 dd (10.50, 3.18)	6.71 dd (10.60, 2.92)	6.75 dd (10.50, 2.30)	6.74 dd (10.45 2.10)	6.41 dd (10.56, 3.18)
13					
14					
15	2.27 m	2.31 m	2.00 m, 1.10 m	2.02 m, 1.06 m	1.72 m, 1.09 m
16a	2.28 m	2.30 dd (14.00, 3.16)	2.28 m	2.28 m	1.98 m
16b	1.82 m	1.83 dt (14.60, 4.52)	1.90 m		1.73 m
17					
18					
19a	3.00 d (14.22)	3.01 d (14.48)	2.86 d (14.50)	2.87 d (14.45)	2.57 d (14.70)
19b	2.64 d (14.70)	2.66 d (14.64)	2.28 m	2.29 m	1.70 m
20					
21			4.11 dd (11.85, 4.15)	4.11 dd (10.70, 3.90)	3.51 dd (12.36, 4.56)
22a	3.33 d (14.70)	3.34 d (14.64)	3.11 dd (12.90, 4.15)	3.11 dd (12.85, 4.10)	2.44 dd (12.78, 4.56)
22b	2.58 m	2.59 d (14.68)	1.83 m	1.83 m	1.33 m
23	1.16 s	1.26 s	1.15 s	1.21 s	0.95 s
24	1.01 s	1.04 s	1.00 s	0.88 s	0.82 s
25	0.97 s	0.98 s	0.98 s	1.01 s	0.90 s
26	1.01 s	1.05 s	1.07 s	1.13 s	0.78 s
27	1.10 s	1.14 s	1.07 s	0.98 s	0.97 s
28					
29	1.23 s	1.24 s	1.14 s	1.13 s	0.74 s
30	1.15 s	1.17 s	1.25 s	1.24 s	1.00 s

^{1}H data were measured at [a] 600, [b] 400 and [c] 500 MHz in pyridine-d_5, respectively. [d] in chloroform-*d*.

Table S2. ^{1}H-NMR spectral data of compounds **6**–**9**.

Position	**6** [a]	**7** [b]	**8** [c]	**9** [a]
	δH (*J* in Hz)			
1a	1.86 m	1.75 m	2.61 m	2.62 m
1b		1.33 m	2.41 m	2.42 m
2a	2.33 m	1.97 m	2.20 m	2.19 m
2b	1.90 m			1.76 m
3	3.43 dd (11.94, 4.56)	3.59 br s		
4				
5	0.83 d (11.94)	1.65 m	1.34 m	1.34 m
6	1.56 m, 1.38 m	1.43 m	1.34 m	1.33 m
7	1.24 m	1.45 m, 1.26 m	1.34 m, 1.21 m	1.33 m
8				
9	1.99 s	1.81 m	1.87 d (8.76)	1.86 m
10				
11	5.75 d (10.74)	1.97 m	4.09 m	4.07 m
12	6.58 d (10.56)	5.50 m	5.84 d (3.60)	5.83 m
13				
14				
15a	2.34 m	2.17 m	2.16 m	2.12 m
15b	2.23 m			1.04 m
16a	2.24 m	2.07 m	2.12 m	2.10 m
16b	1.70 m			1.79 m
17				
18		3.57 m	3.64 m	3.67 m
19a	2.80 m	2.16 m	3.14 d (14.36)	3.13 m
19b	2.60 d(14.70)	1.55 dd (14.05, 4.55)	2.57 m	2.55 m
20				
21				
22a	3.30 d (14.22)	3.09 d (14.35)	2.22 m	2.21 m
22b	2.48 d (14.22)	2.56 d (14.35)	1.71 m	1.73 m
23	1.33 s	1.18 s	1.14 s	1.13 s
24	1.00 s	0.87 s	1.05 s	1.04 s
25	0.93 s	0.94 s	1.10 s	1.10 s
26	0.95 s	1.07 s	1.09 s	1.09 s
27	1.12 s	1.02 s	1.07 s	1.04 s
28				
29	1.15 s	1.18 s	1.23 s	1.17 s
30	1.12 s	1.05 s	1.19 s	1.22 s
1′	4.95	6.20 d (8.15)	6.22 d (8.12)	6.21 m
2′	4.06 m	4.08 m	4.29 m	4.28 m
3′	4.26 m	4.17 t (8.90)	4.20 m	4.18 m
4′	4.24 m	4.24 t (9.10)	4.27 m	4.26 m
5′	4.04 m	4.09 m	3.88 m	3.86 m
6′a	4.61 m	4.66 d (10.50)	4.67 m	4.66 m
6′b	4.43 dd (11.46, 5.04)	4.31 m		4.26 m
1″		4.94 d (7.80)	4.93 m	4.91 m
2″		3.92 t (8.05)	3.91 m	3.90 m
3″		4.06 m	3.63 m	3.61 m
4″		4.07 m	4.35 m	4.33 m
5″		3.80 m	4.09 m	4.08 m
6″a		4.61 m	4.18 m	4.14 m
6″b		4.51 m	4.05 m	4.02 m
1‴		5.51 m	5.81 m	5.78 m
2‴		4.60 m	4.52 dd (9.20, 3.28)	4.49 m
3‴		4.48 m	4.64 m	4.62 m

Table S2. *Cont.*

Position	6 [a]	7 [b]	8 [c]	9 [a]
	δH (*J* in Hz)			
4‴		4.31 m	4.09 m	4.08 m
5‴		4.84 m	4.91 m	4.88 m
6‴		1.69 d (6.15)	1.68 d (6.20)	1.65 d (5.94)
OCOCH3		1.89 s		
OMe			3.25 s	
OBu				3.56 m, 3.24 m
				1.47 m, 1,33 m
				1.32 m
				0.81 m

^{1}H data were measured at [a] 600, [b] 500 and [c] 400 MHz in pyridine-d_5, respectively.

Table S3. ^{13}C-NMR spectral data of compounds **1**–**5**.

Position	1 [a]	2 [b]	3 [c]	4 [c]	5 [a,d]
	δC				
1	38.6	38.4	38.7	33.4	32.7
2	34.1	28.0	34.1	26.4	25.2
3	216	78.0	216.0	75.3	76.2
4	47.5	39.5	47.5	38.1	37.5
5	54.3	55.2	54.3	48.8	48.5
6	19.6	18.7	19.7	18.6	18.2
7	31.8	32.7	31.9	32.8	32.2
8	41.0	41.2	40.8	41.3	41.0
9	53.9	54.8	54.0	54.7	54.2
10	36.5	37.1	36.6	37.4	36.9
11	128.2	128.9	126.6	127.5	128.1
12	125.9	125.5	126.4	126.0	124.9
13	138.8	139.1	136.7	137.0	138.0
14	42.3	42.4	42.4	42.5	42.1
15	26.0	26.0	25.6	25.6	24.8
16	31.9	32.0	33.2	33.3	32.4
17	51.0	51.0	49.6	49.7	48.8
18	129.6	129.2	132.7	132.2	ND
19	40.7	40.4	40.7	40.7	39.8
20	46.1	46.1	37.8	37.8	37.0
21	212.2	212.5	73.9	74.0	74.4
22	49.7	49.7	44.5	44.7	42.8
23	26.4	28.5	26.4	29.2	28.0
24	20.9	16.0	20.9	22.1	21.7
25	17.5	18.4	17.5	18.3	17.9
26	16.5	16.9	16.6	17.1	16.4
27	20.1	20.3	20.0	20.0	19.9
28	177.8	177.9	178.9	179.1	178.9
29	24.8	24.9	18.1	18.1	17.1
30	25.5	25.6	29.0	29.1	28.1

^{13}C data were measured at [a] 600, [b] 400 and [c] 500 MHz in pyridine-d_5, respectively. [d] in chloroform-*d*.

Table S4. ^{13}C-NMR spectral data of compounds **6**–**9**.

Position	**6** [a]	**7** [b]	**8** [c]	**9** [a]
	δC			
1	38.1	33.6	34.6	34.5
2	26.3	26.3	40.4	40.4
3	88.8	75.1	216.3	216.3
4	39.5	37.8	47.7	47.7
5	55.2	49.2	55.5	55.5
6	18.3	18.6	19.9	19.6
7	32.6	33.0	32.9	32.8
8	41.0	37.5	38.0	38.0
9	54.6	47.8	52.1	52.6
10	36.6	41.9	43.0	43.0
11	127.5	23.8	76.4	76.3
12	126.0	124.4	124.0	124.2
13	137.6	141.8	146.1	145.6
14	42.2	40.0	42.1	42.0
15	26.5	28.0	28.1	28.0
16	32.1	25.8	25.6	25.6
17	50.2	45.5	45.4	45.4
18	132.2	41.2	40.9	40.8
19	40.5	47.5	46.2	46.2
20	46.0	51.1	50.9	50.8
21	213.8	212.4	212.0	212.0
22	50.4	46.5	47.2	47.2
23	27.9	29.3	26.5	26.4
24	16.5	22.7	21.5	21.4
25	18.3	15.5	16.5	16.5
26	16.9	17.5	18.9	18.9
27	20.2	26.0	25.3	25.0
28	185.0	173.9	174.0	173.9
29	24.8	24.6	24.6	24.5
30	25.7	25.2	25.0	25.2
1′	107.0	96.0	96.1	96.0
2′	75.7	76.3	74.0	73.8
3′	78.7	78.6	78.6	78.5
4′	71.8	70.9	70.8	70.7
5′	78.3	78.0	76.1	75.1
6′	63.0	69.5	69.4	69.3
1″		104.9	105.0	104.9
2″		75.0	75.2	74.6
3″		73.8	77.1	77.0
4″		79.2	78.3	78.2
5″		73.7	78.0	77.9
6″		63.6	61.3	61.2
1‴		102.9	102.7	102.6
2‴		72.3	72.7	72.6
3‴		72.6	72.5	72.4
4‴		73.7	73.7	73.6
5‴		70.6	70.3	70.2
6‴		18.5	18.5	18.4

Table S4. *Cont.*

Position	6 [a]	7 [b]	8 [c]	9 [a]
	δC			
OCOCH3		170.5		
OCOCH3		20.6		
OMe			54.4	
OBu				66.7
				32.8
				19.8
				13.9

^{13}C data were measured at [a] 600, [b] 500 and [c] 400 MHz in pyridine-d_5, respectively.

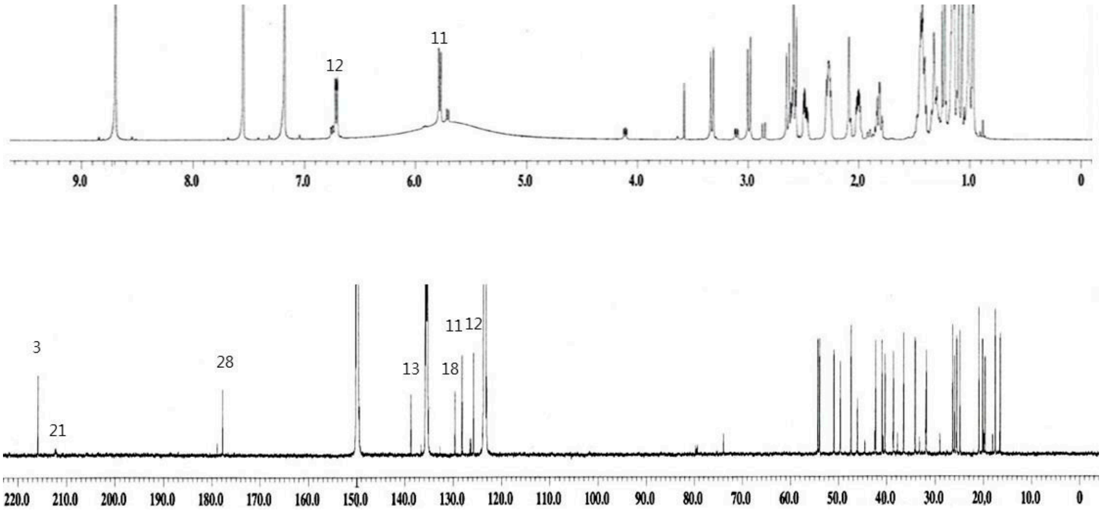

Figure S1. ^{1}H- and ^{13}C-NMR spectra of compound **1**.

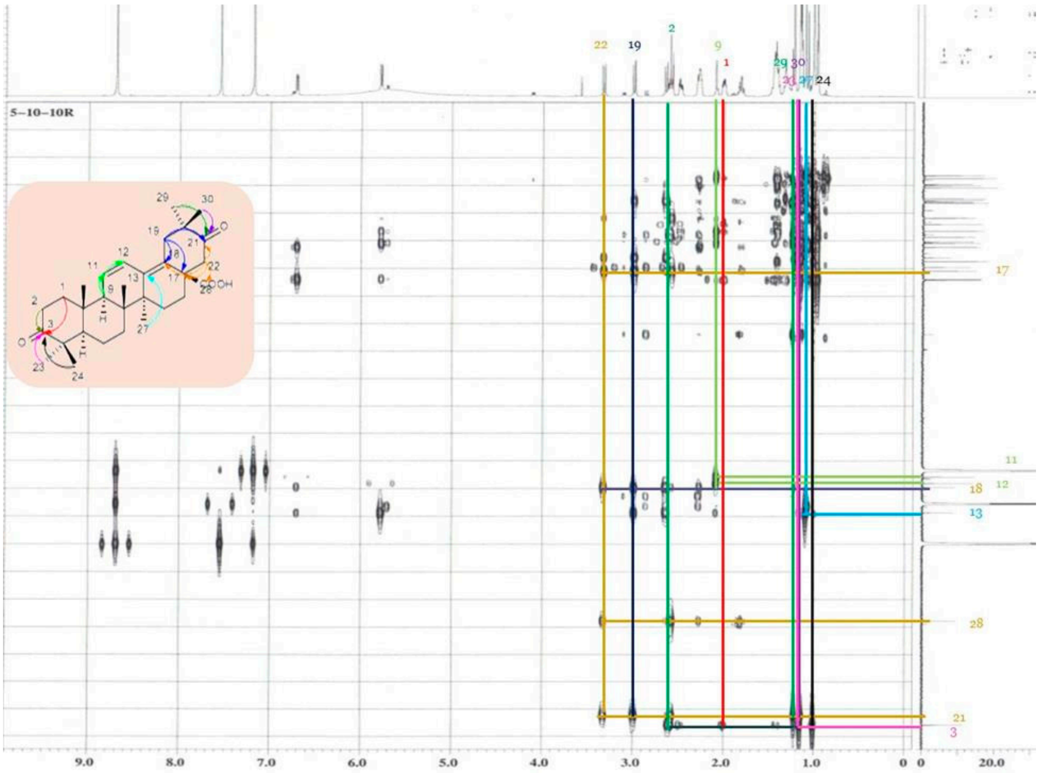

Figure S2. ^{1}H- and ^{13}C-NMR spectra of compound **1**.

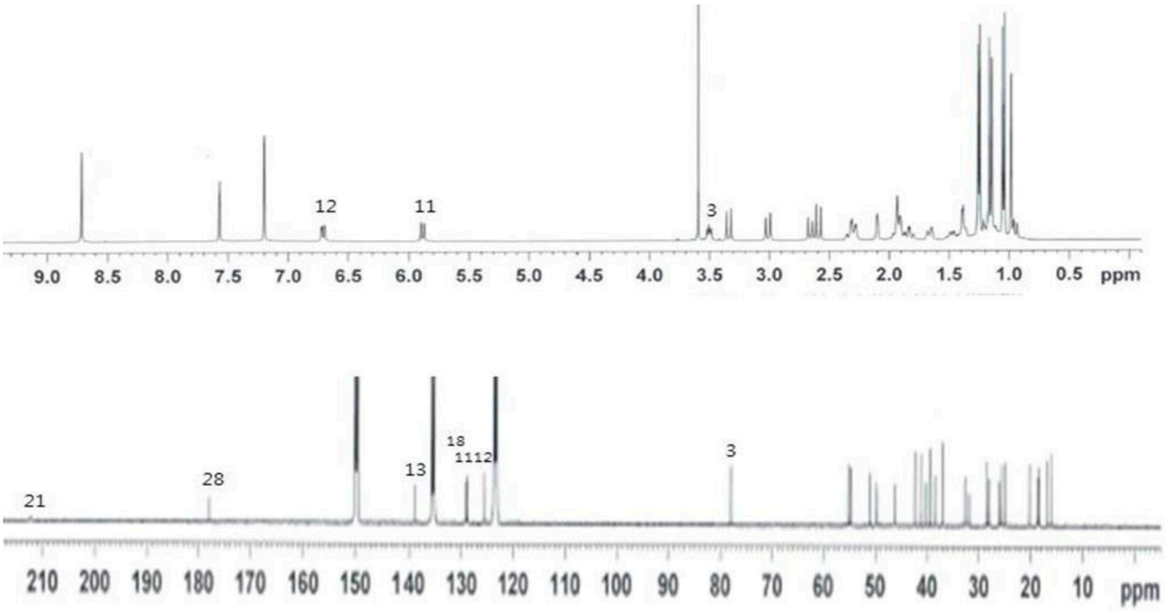

Figure S3. ^{1}H- and ^{13}C-NMR spectra of compound **2**.

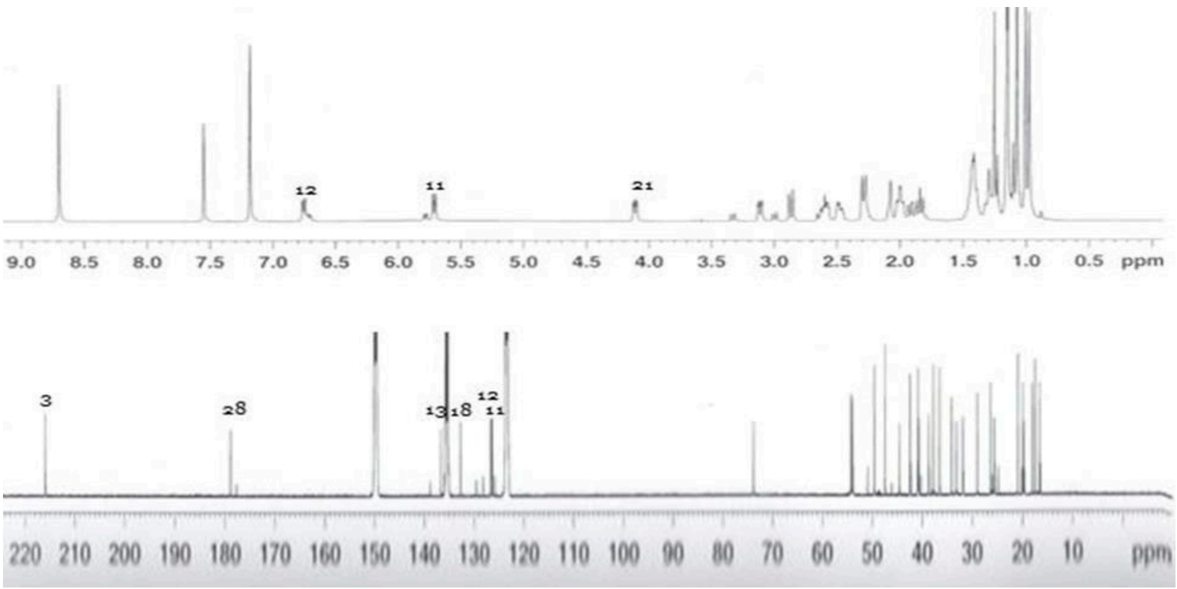

Figure S4. ^{1}H- and ^{13}C-NMR spectra of compound **3**.

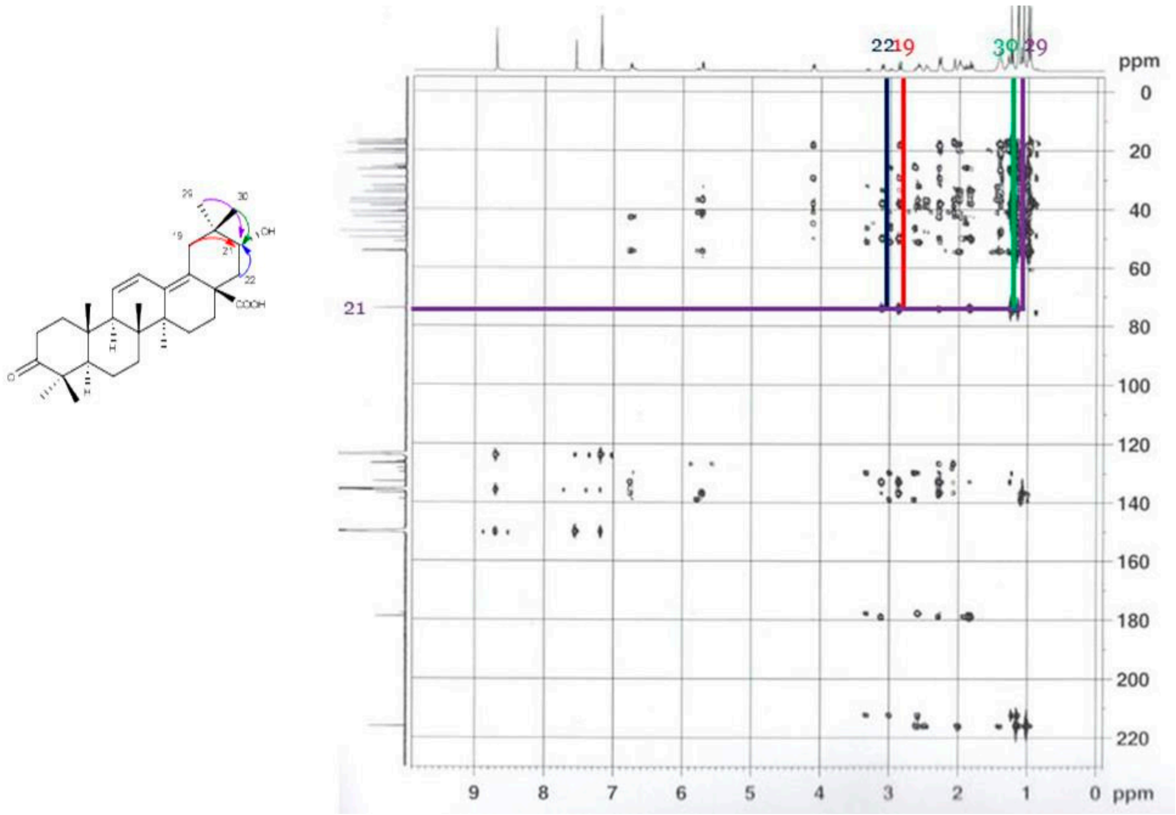

Figure S5. HMBC spectrum of compound **3**.

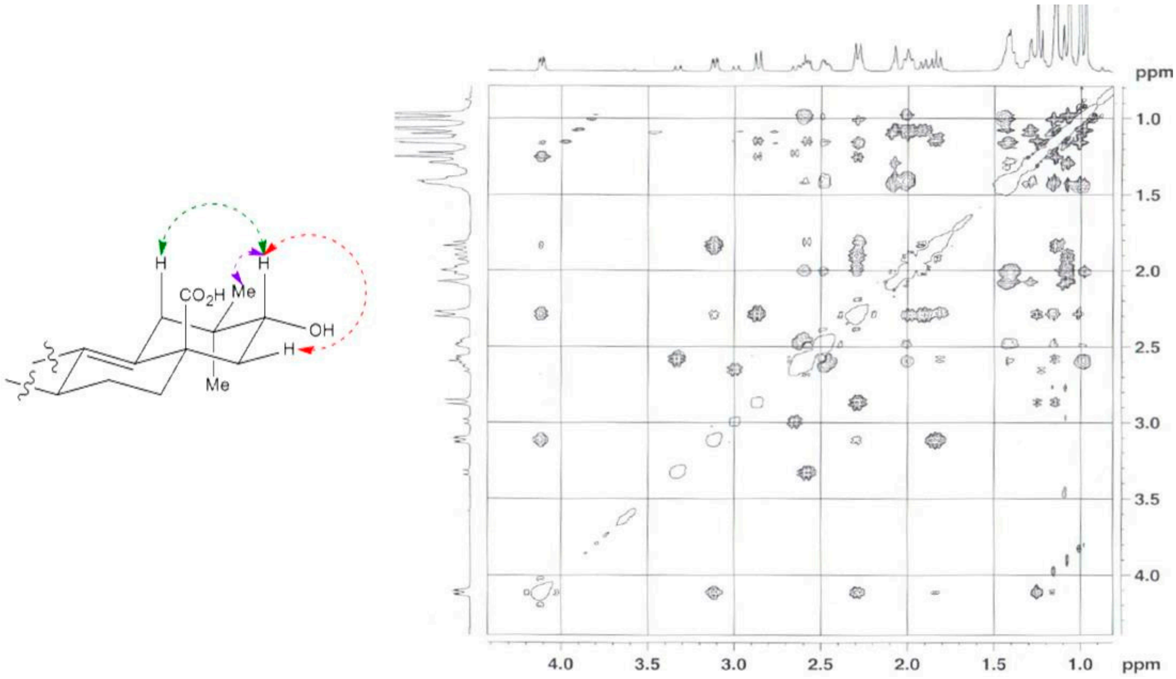

Figure S6. NOESY spectrum of compound **3**.

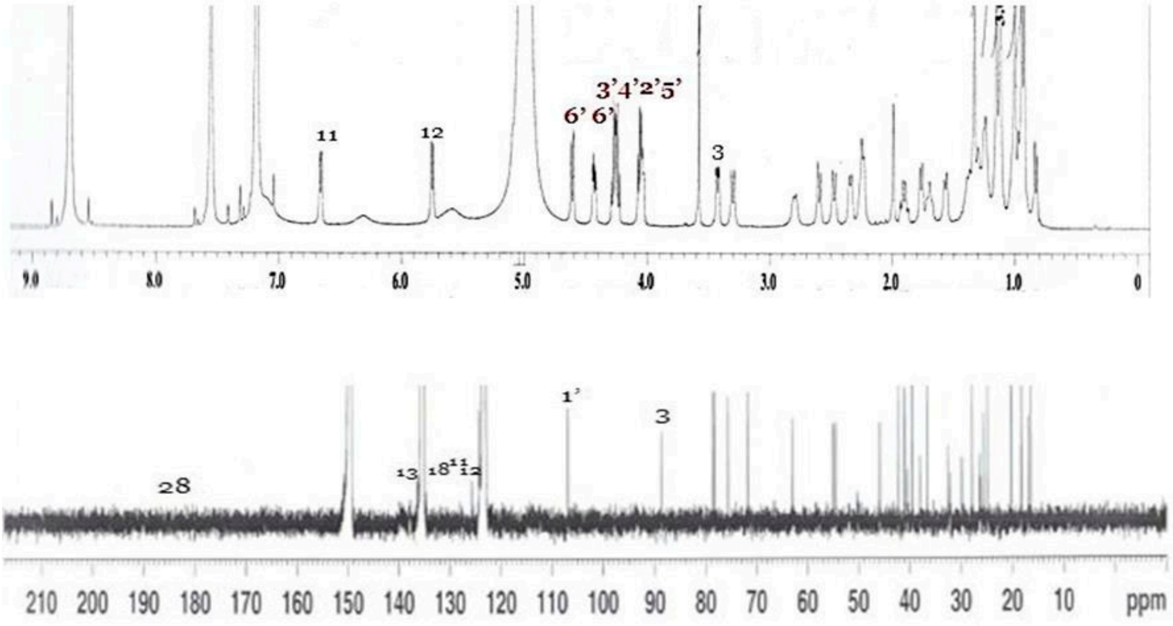

Figure S7. ^{1}H- and ^{13}C-NMR spectra of compound **6**.

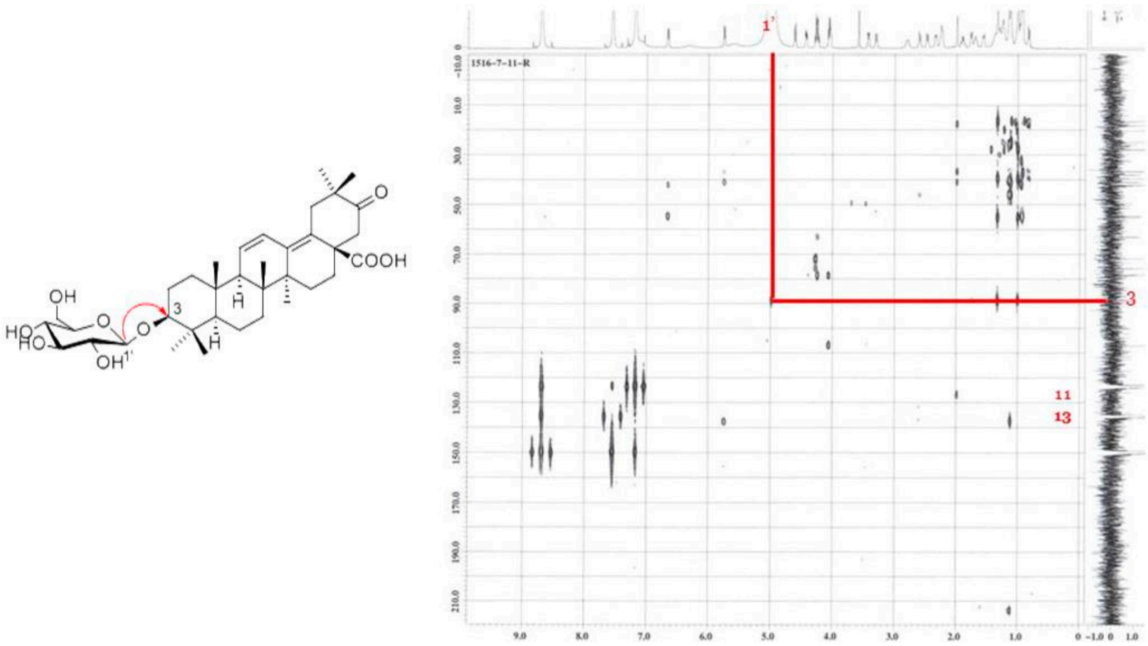

Figure S8. HMBC spectrum of compound **6**.

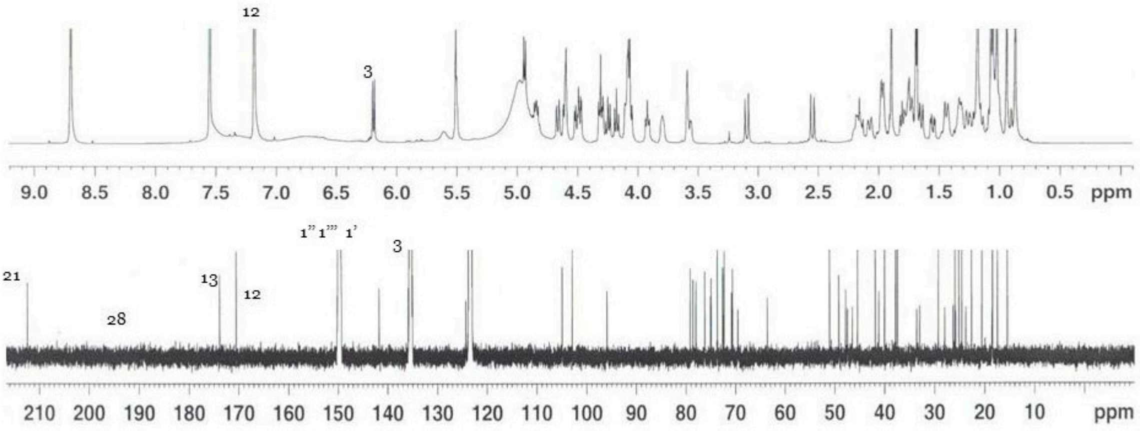

Figure S9. ^{1}H- and ^{13}C-NMR spectra of compound **7**.

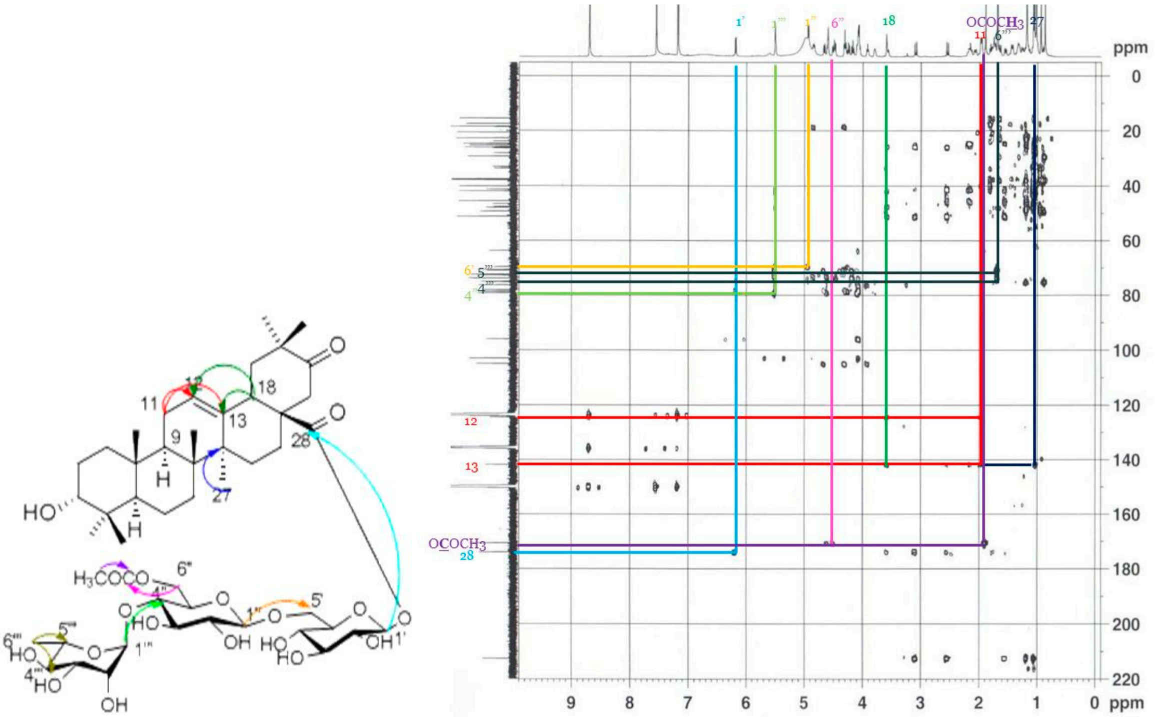

Figure S10. HMBC spectrum of compound **7**.

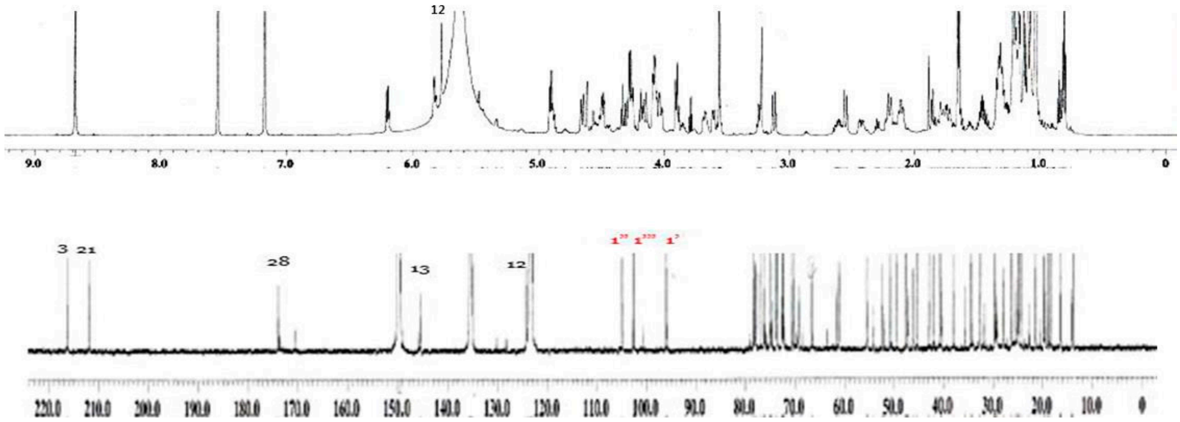

Figure S11. ^{1}H- and ^{13}C-NMR spectra of compound **9**.

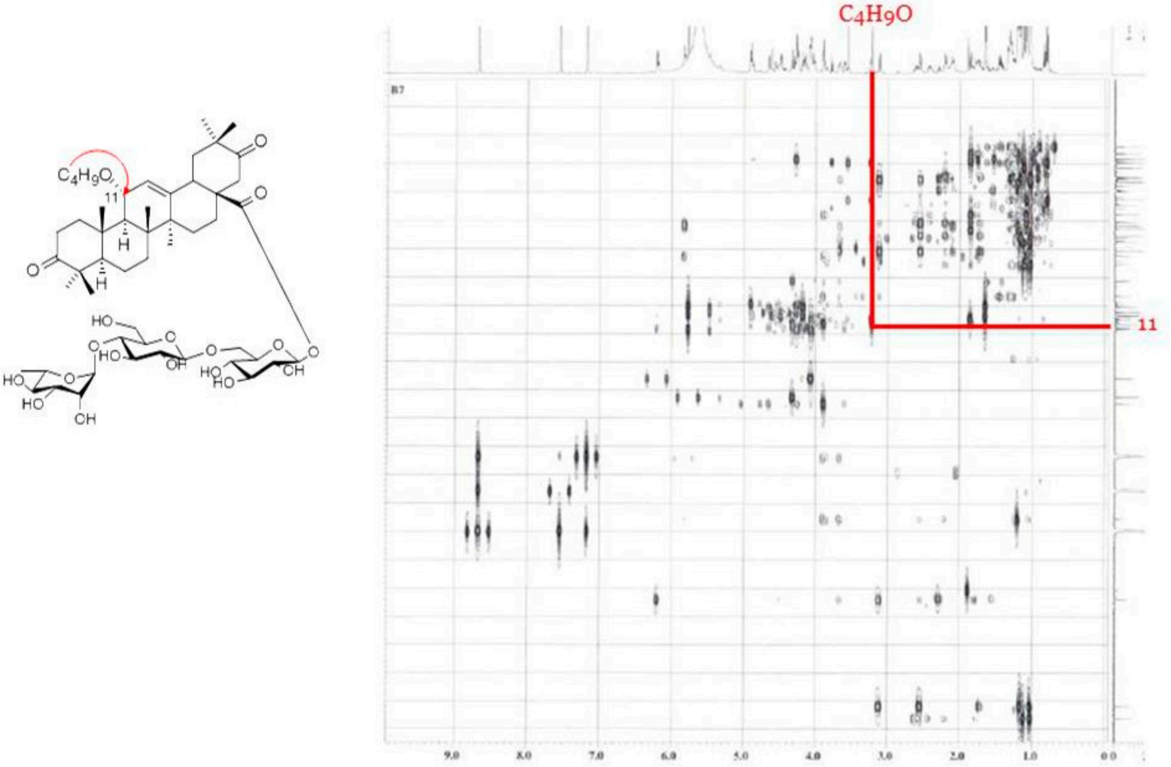

Figure S12. HMBC spectrum of compound **9**.

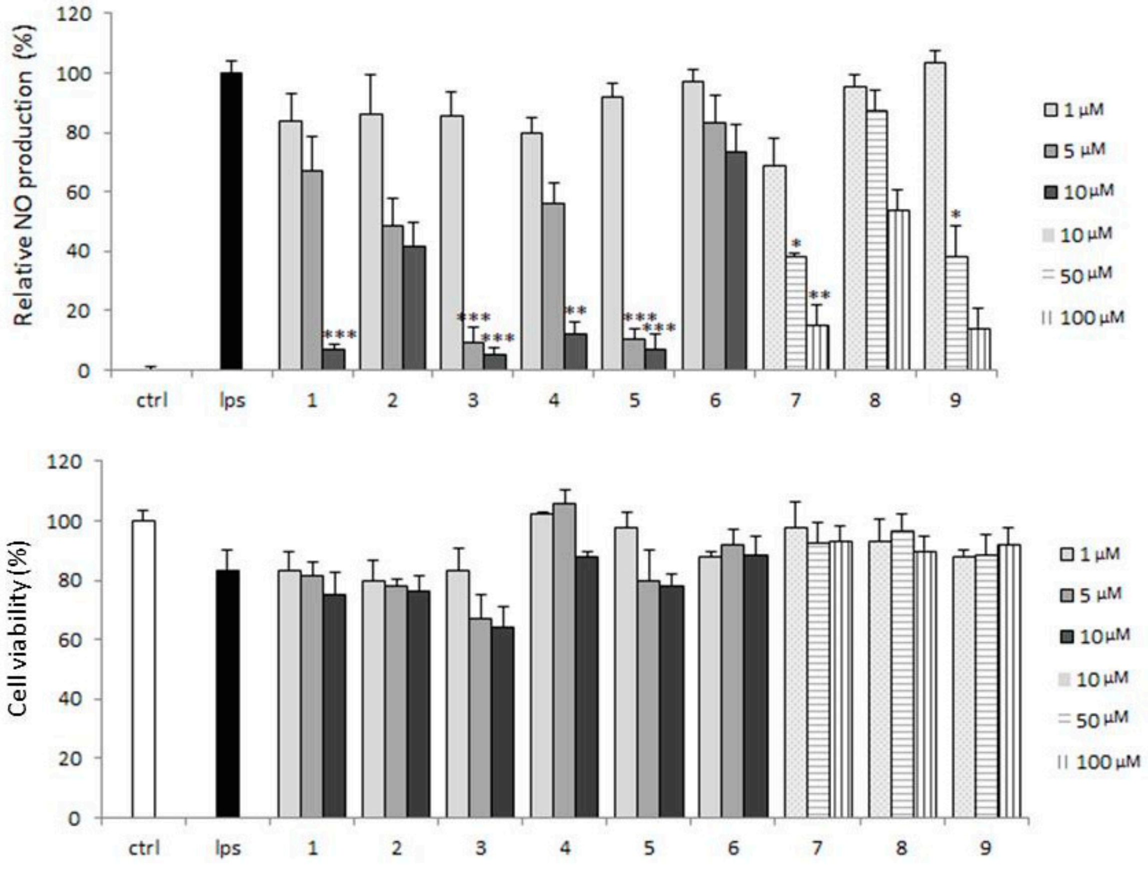

Figure S13. NO inhibitory effects of compounds **1**–**9** isolated from *T. papyriferus* on LPS-activated BV2 microglia cells.
